# Dinoflagellate responses to nutrients and mangrove leaf organic matter in the bioluminescent Mangrove Lagoon, St. Croix, U.S. Virgin Islands

**DOI:** 10.1111/jpy.70189

**Published:** 2026-06-17

**Authors:** James L. Pinckney, Michelle Zimberlin, Dianne I. Greenfield

**Affiliations:** ^1^ Department of Biological Sciences University of South Carolina Columbia South Carolina USA; ^2^ School of the Earth, Ocean, and Environment University of South Carolina Columbia South Carolina USA; ^3^ Advanced Science Research Center City University of New York New York New York USA; ^4^ Queens College, City University of New York New York New York USA

**Keywords:** ChemTax, eutrophication, loading, phytoplankton, *Pyrodinium*

## Abstract

Bioluminescent bays (biobays) are uncommon coastal ecosystems featuring dense populations of dinoflagellates, often found in mangrove‐ringed lagoons with long water retention times and high organic matter (OM) levels. Although mangroves are associated with high dinoflagellate abundance, it remains uncertain how inorganic nutrients compare with mangrove‐derived organic matter in shaping phytoplankton communities. We performed short‐term in situ bioassays in Mangrove Lagoon, St. Croix, U.S. Virgin Islands, to compare phytoplankton community responses to additions of inorganic nutrients (N and P) and senescent mangrove leaf additions. Phytoplankton composition was assessed using high‐performance liquid chromatography (HPLC) pigment analysis combined with CHEMTAX, and data were analyzed with compositional and centered log‐ratio principal component analyses (CLR–PCAs). Nutrient additions primarily increased total phytoplankton biomass and favored diatom‐dominated communities, consistent with N limitation, but did not significantly alter community composition. Conversely, adding senescent mangrove leaves consistently shifted the community toward higher abundances of peridinin‐containing dinoflagellates, without significantly increasing the abundance of other phytoplankton groups. Multivariate analysis showed that leaf additions shifted the community along a continuous compositional spectrum rather than creating distinct assemblages. These findings suggest that OM (leachate) from senescent mangrove leaves, rather than inorganic N and P enrichment alone, is a plausible mechanism for promoting dinoflagellate dominance in Mangrove Lagoon. Our results underscore the potential role of mangrove OM input in sustaining dinoflagellate‐dominated environments in some tropical lagoons, demonstrating a mechanism by which mangroves may regulate phytoplankton community composition in adjacent waters.

**Abbreviations**
ANOVA: analysis of variance
CLR‐PCA: centered log‐ratio principal components analysis
DOC: dissolved organic carbon
DOM: dissolved organic matter
DON: dissolved organic nitrogen
HPLC: high‐performance liquid chromatography
OM: organic matter
PCA: principal component analysis
PERMANOVA: permutational multivariate analysis of variance
SARI: Salt River Bay

## INTRODUCTION

Bioluminescent bays (“biobays”) are rare coastal ecosystems characterized by persistent, high‐density populations of dinoflagellates. Beyond their aesthetic, cultural, and ecotourism value, biobays are ecologically significant for their atypical nutrient regimes, long water residence times, and elevated concentrations of dissolved organic matter (DOM). These conditions favor dinoflagellate taxa, including potentially toxic species (Phlips et al., 2006; Seliger et al., 1970, 1971). In the Caribbean, biobays are associated with dense populations of the dinoflagellate *Pyrodinium bahamense* var. *bahamense* and other bioluminescent species (Cusick et al., 2024; Morquecho, 2019; Seliger et al., 1970; Usup et al., 2012). Although *P. bahamense* was previously considered non‐toxic in North America, recent studies documented saxitoxin production in *P. bahamense* in the Caribbean and the Indian River Lagoon, Florida, indicating that this species is also a growing public health threat (Cusick et al., 2024; Shankar et al., 2023; Usup et al., 2012). Blooms of *P. bahamense* and other bioluminescent dinoflagellate species have been documented in shallow lagoons and semi‐enclosed coastal systems throughout the Caribbean, Gulf of Mexico, Gulf of California, and Florida, where low flushing rates and restricted circulation promote the accumulation of plankton biomass (Martínez‐Hernández & Hernández‐Campos, 1991; Morquecho, 2019; Peña‐Manjarrez et al., 2005; Usup et al., 2012). Short‐term introduction of low‐salinity waters to these systems, particularly following severe storms, is also associated with higher abundances of *P. bahamense* (Govender & Jury, 2024; Phlips et al., 2006), suggesting that these events may supply nutrients or other inputs to biobays that favor dinoflagellates over co‐occurring phytoplankton species.

A defining physical and ecological feature of many biobays is their close association with extensive mangrove forests, particularly the red mangrove (*Rhizophora mangle*; Alongi, 2014). Upon immersion, senescent mangrove leaves release DOM, inorganic nutrients, and a complex suite of secondary metabolites into surrounding waters (Benner et al., 1986; Hernes et al., 2001; Phlips et al., 2006; Zhang & Laanbroek, 2018; Zhou et al., 2023). As a result, mangrove‐fringed lagoons frequently exhibit elevated dissolved organic carbon (DOC) concentrations, altered nutrient stoichiometry, and enhanced microbial activity relative to adjacent coastal waters (Benner et al. 1986; Benner, Hatcher, et al., 1990; Benner, Weliky, et al., 1990; Zhou et al., 2023; Yang et al., 2024). Thus, mangrove‐derived inputs may play a central role in sustaining dinoflagellate‐dominated phytoplankton assemblages in biobays (Phlips et al., 2006; Usup et al., 2012; Zhou et al., 2023). Several mechanisms have been proposed, including direct nutrient enrichment, stimulation of microbial regeneration pathways, provision of organic substrates that support mixotrophic growth, and selective inhibition of competing phytoplankton groups by mangrove‐derived phenolic compounds, tannins, or humic substances (Alongi, 2014; Benner et al., 1986; Maie et al., 2008; Zhang & Laanbroek, 2018; Zhou et al., 2023). However, the specific drivers linking mangrove inputs to dinoflagellate populations remain poorly resolved.

Dinoflagellates have a suite of physiological and ecological traits that may confer competitive advantages in organic‐rich, low‐turbulence environments (Margalef, 1978; Reynolds, 2006). Many taxa exhibit flexible nutritional strategies, including mixotrophy and phagotrophy, enabling them to exploit both inorganic nutrients and organic carbon sources unavailable to autotrophic phytoplankton, such as diatoms (Jeong et al., 2010; Millette et al., 2023; Stoecker, 1999). In addition, dinoflagellates often tolerate low‐nutrient concentrations, stratification, and reduced light (Margalef, 1978; Reynolds, 2006).

Mangrove Lagoon, within the Salt River Bay National Historical Park and Ecological Preserve on St. Croix, U.S. Virgin Islands, is a compelling system for examining phytoplankton community dynamics. This shallow, human‐made embayment exhibits consistent, year‐round dinoflagellate bioluminescence, indicating persistent ecological conditions favoring high relative abundances of dinoflagellates. Previous observations showed that the lagoon has low water exchange, elevated DOC concentrations, and dissolved inorganic nutrient ratios suggestive of nitrogen (N) limitation (Pinckney et al., 2018; Reidhaar et al., 2016; Zimberlin, 2013). Together, these characteristics make Mangrove Lagoon an ideal natural laboratory for disentangling the relative roles of inorganic nutrients and senescent mangrove leaf leachate in structuring phytoplankton communities.

Although correlations between mangroves and dinoflagellate abundance are well documented, experimental tests that isolate the effects of nutrients from those of mangrove‐derived DOM remain limited (Phlips et al., 2006; Seliger et al., 1970, 1971). Few studies have directly compared community‐level phytoplankton responses to inorganic nutrient enrichment and whole‐leaf mangrove additions under in situ conditions. Such experiments are necessary to determine whether mangrove leaf inputs primarily alleviate nutrient limitation, restructure competitive interactions within the phytoplankton community, or selectively promote dinoflagellates through pathways mediated by organic matter (OM).

The purpose of this study was to experimentally evaluate acute phytoplankton community responses to inorganic nutrient additions (nitrogen, N and phosphorus, P) and senescent mangrove leaf additions using short‐term in situ bioassays in Mangrove Lagoon. We focused on changes in relative phytoplankton group composition, with particular attention to dinoflagellates, using pigment‐based chemotaxonomic estimates of community composition coupled with compositional data analysis. By comparing the effects of nutrient enrichment and mangrove leaf leachate on community structure, this study provides empirical insight into why dinoflagellates, including the bioluminescent *Pyrodinium bahamense*, achieve high and persistent abundances in some Caribbean mangrove lagoons.

## MATERIALS AND METHODS

### Study site

**FIGURE 1:**
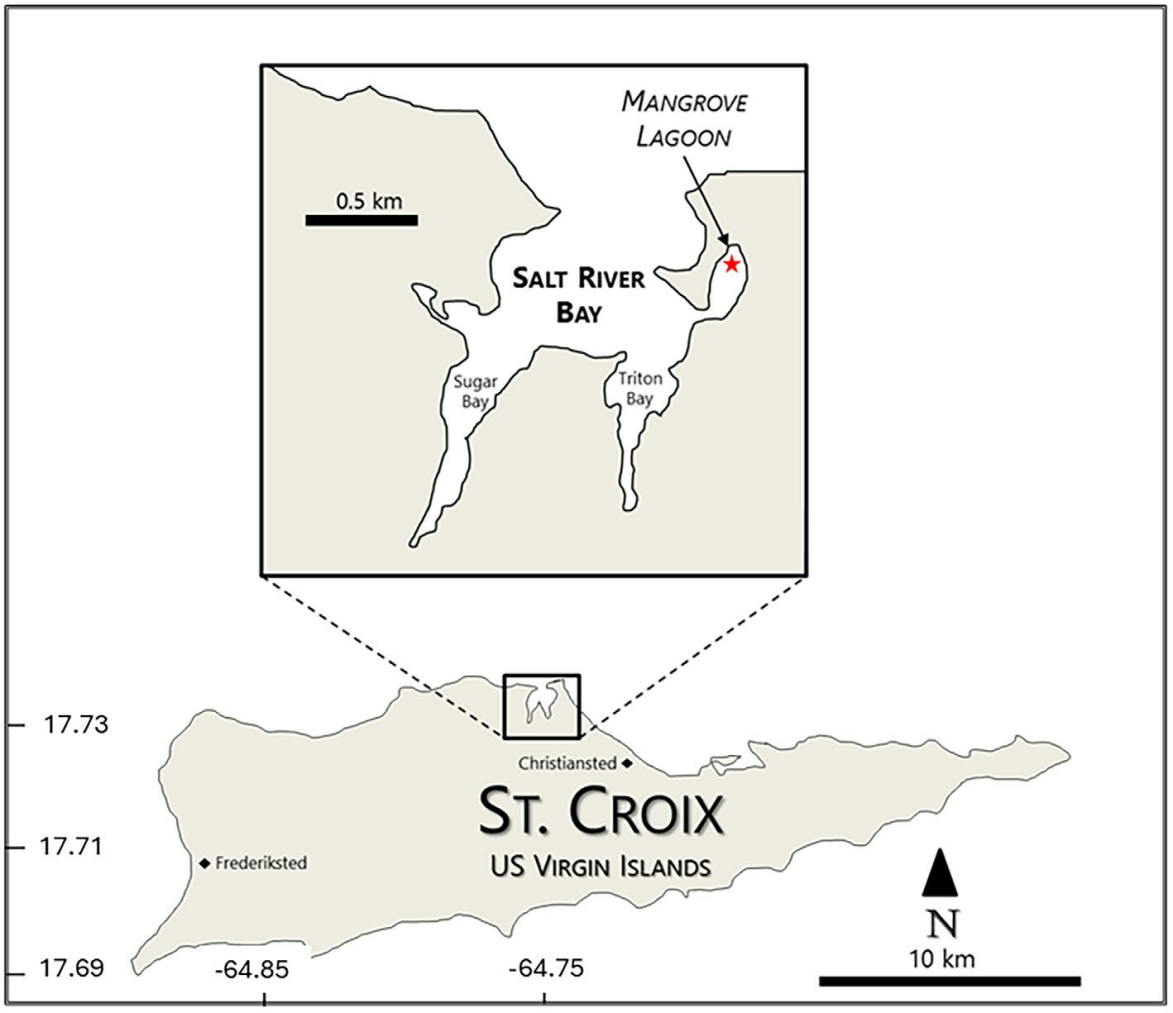
Location map for the study site, Mangrove Lagoon, St. Croix, U.S. Virgin Islands. The red star indicates where incubation samples were collected.

St. Croix, the largest of the U.S. Virgin Islands, lies 151 km southeast of Puerto Rico (17.73° N, 64.75° W; Figure 1). The island is 39 km long and 9 km wide, with a total area of 207 km^2^. Salt River Bay (SARI) is a 4.10‐km^2^ National Park established in 1992 that encompasses diverse habitats, including mangroves, seagrass beds, reefs, and submarine canyons. Mangrove Lagoon is a small (250 m × 130 m), shallow (<4 m), human‐made embayment on the east side of SARI (17.79° N, 64.75° W), which is connected to SARI via a narrow channel that limits water exchange with the larger bay (Figure 1). Salinities in the lagoon range from 29 to 41, with an annual mean of 37 ± 2.0 *SD*, and the average annual water temperature is 29 ± 2°C *SD* (Pinckney et al., 2018). The bottom sediments consist of calcium carbonate, sand, and fine silt (Reidhaar et al., 2016). In this lagoon, dissolved inorganic nitrogen (DIN) ranges from 0.4 to 8.4 μM (annual mean = 4.0 ± 1.9 μM *SD*), while concentrations of dissolved inorganic phosphorus (DIP) average 0.7 ± 0.1 μM (range = 0.5–0.9 μM; Zimberlin, 2013; Pinckney et al., 2018). The N:P molar ratio for Mangrove Lagoon (annual mean = 6.5 ± 3.0 *SD*) suggests N limitation for the phytoplankton community based on the Redfield ratio of 16 N:1 P, and this was confirmed by Zimberlin (2013). The waters of Mangrove Lagoon are unique because they are one of a few biobays in the Caribbean that exhibit year‐round dinoflagellate bioluminescence (Pinckney et al., 2018).

### Bioassays

In situ bioassays with nutrient and leaf additions were conducted in January and May 2013. The water used in the bioassays was collected from the northeastern corner of Mangrove Lagoon, which has historically exhibited the highest intensity of nighttime bioluminescence and the highest concentrations of dinoflagellate cysts in the sediments (Pinckney et al., 2018; Reidhaar et al., 2016). Water from the upper 1 m of the water column was collected around 10 a.m. local time using an integrated water sampler (8‐cm‐diameter PVC baler) and dispensed into acid‐washed 1‐L clear polycarbonate Nalgene flasks. For bioassays, nutrient additions were 10 μM NO_3_^−^ (N), 5 μM PO_4_^−3^ (P), N + P (NP), and there was a control (no nutrients added). Each treatment had eight replicates.

Water for the mangrove leaf addition bioassays was collected and dispensed into bottles as described above. Senescent orange and yellow leaves were randomly collected from red mangrove (*Rhizophora mangle*) trees surrounding the lagoon and placed into bioassay bottles along with water from the lagoon. Six senescent mangrove leaves were placed in each flask, with 16 replicates, such that each replicate received a random assortment of orange and yellow leaves.

The incubation flasks were returned to the lagoon's surface waters and placed within a floating mesh corral covered with a neutral‐density screen to reduce irradiance by ~40%. Samples collected for the initial phytoplankton community composition were designated as time 0 (*T*_0_). The samples were then incubated for 48 h under ambient conditions. After the experiment, treatment bottles were transported in the dark and in coolers to the University of the Virgin Islands laboratory for subsequent processing. Aliquots (50–250 mL) of the incubation water from the nutrient and leaf bioassays were filtered under gentle vacuum (<50 kPa) through a glass fiber filter (0.7‐μm nominal pore size, 25 mm dia., Whatman GF/F), immediately frozen, and stored at −80°C. The filters and filtrate were then shipped to the University of South Carolina laboratory (Columbia, South Carolina, United States) in a dry shipper cooled with liquid N_2_.

### Analytical methods

Phytoplankton photopigment concentrations were measured using high‐performance liquid chromatography (HPLC; Pinckney et al., 2001). Filters were lyophilized for 18–24 h at −50°C, extracted with 750 μL of 90% aqueous acetone, and stored in the dark for 12–20 h at −20°C. Filtered extracts (250 μL) were injected into a Shimadzu HPLC with a single monomeric column (Rainin Microsorb, 0.46 × 1.5 cm, 3 μm packing) and a polymeric (Vydac 201TP54, 0.46 × 25 cm, 5 μm packing) reverse‐phase C18 column in series. A non‐linear binary gradient consisting of solvent A (80% methanol: 20% 0.5 M ammonium acetate) and solvent B (80% methanol:20% acetone) was used for the mobile phase (Pinckney et al., 1996). Absorption spectra and chromatograms (440 ± 4 nm) were acquired using a Shimadzu SPD‐M10AV photodiode‐array detector, and pigment peaks were identified by comparing retention times and absorption spectra with those of pure standards (DHI, Denmark). The synthetic carotenoid trans‐ β‐apo‐8′‐carotenal (Sigma‐Aldrich) was used as an internal standard.

CHEMTAX (version 1.95) was used to estimate the relative concentrations of major phytoplankton groups from photopigment concentrations (Higgins et al., 2011; Pinckney et al., 2001). This method quantifies the relative contributions of chlorophyll *a* to algal groups, including diatoms, green algae (all chlorophyll *b*‐containing taxa: chlorophytes, euglenophytes, prasinophytes), cryptophytes, cyanobacteria, haptophytes, and two categories of dinoflagellates (peridinin‐containing—type I—and fucoxanthin‐containing—type II—taxa). To minimize errors in biomass estimates due to inaccurate initial pigment ratios, we applied the initial‐ratio matrix randomization technique with 60 matrices (Higgins et al., 2011). The presence of algal taxonomic groups was confirmed by qualitative microscopy at 400× magnification of randomly selected Lugol's‐fixed samples (2% final volume) using settling chambers and an inverted microscope.

Dissolved organic carbon was measured with a Shimadzu TOC‐V analyzer attached to an ASI‐V autosampler. Filtrate (0.7‐μm gf/f filtered) was analyzed for orthophosphate (PO43‐), dissolved nitrate/nitrite (NO_2_^−^/NO_3_^−^), and ammonium (NH_4_^+^) using a Lachat Quick‐Chem 8000 nutrient auto‐analyzer according to standard methods (Grasshoff et al., 1999; Johnson & Petty, 1983; Zimmerman & Keefe, 1991).

### Statistics

Specific treatment effects on individual phytoplankton groups were evaluated using nonparametric Kruskal‐Wallis analysis of variance (ANOVA) tests to compare leaf and nutrient additions. Phytoplankton community composition was further analyzed using relative abundance data, expressed as the proportion of total chlorophyll *a* attributed to major phytoplankton taxonomic groups (cyanobacteria, cryptophytes, dinoflagellates types I & II, green algae, diatoms, haptophytes). The response variables were compositional because the proportions within each sample (i.e., ratio of treatment to control) summed to a constant (i.e., 1.00). Compositional data analysis techniques were used, rather than conventional multivariate methods based on raw percentages, to appropriately analyze such data (Aitchison, 1986). A small pseudo‐count (0.001) was added to all variables to enable log‐ratio transformation. Data were then transformed using the centered log‐ratio (CLR) transformation, which removes the constant‐sum constraint and enables Euclidean geometry (Aitchison, 1986; Egozcue et al., 2003).

A permutational multivariate analysis of variance (PERMANOVA; 4999 permutations, fixed random seed 2026) was performed on the CLR‐transformed data to test for treatment effects on community composition. Effect size (*R* ^2^) and confidence intervals were estimated using case bootstrap resampling (2000 bootstrap replicates, 95% percentile interval). Post hoc comparisons between treatments, using permutation tests and the Benjamini‐Hochberg (false discovery rate, FDR) correction, were applied for multiple comparisons (Benjamini & Hochberg, 1995).

Principal component analysis (PCA) was then performed on the CLR‐transformed data to identify dominant gradients in relative community structure (Jolliffe, 2002). The PCA scores, loadings, and proportion of variance explained by each axis were obtained using the PCA application in the scikit‐learn Python library (Pedregosa et al., 2011). Ordination results were visualized by plotting sample scores on the first two principal components and overlaying rescaled loading vectors to illustrate the contribution of individual phytoplankton groups to each axis. All analyses were conducted in Python using the pandas, numpy, scikit‐learn, and matplotlib libraries (Hunter, 2007; McKinney, 2010; Oliphant, 2006).

## RESULTS

For the January incubations, inorganic N and P concentrations in the ambient seawater were 2.31 and 0.50 μM, respectively. For May, the concentrations were 1.10 μM N and 0.50 μM P. The ambient N:P atomic ratios for January and May were 5:1 and 2:1, respectively, consistent with N limitation under short‐term enrichment conditions. The nutrient additions of 10 μM NO_3_^−^ and 5 μM PO_4_^−3^ were thus significant relative to ambient concentrations and likely minimized the effects of nutrient limitation in the 48‐h bioassays. For the mangrove leaf bioassays, ambient DOC concentrations were 79 and 212 μM C for the January and May experiments, respectively. Each leaf added an average of 222 μM C per leaf in January and 553 μM C in May.

**FIGURE 2:**
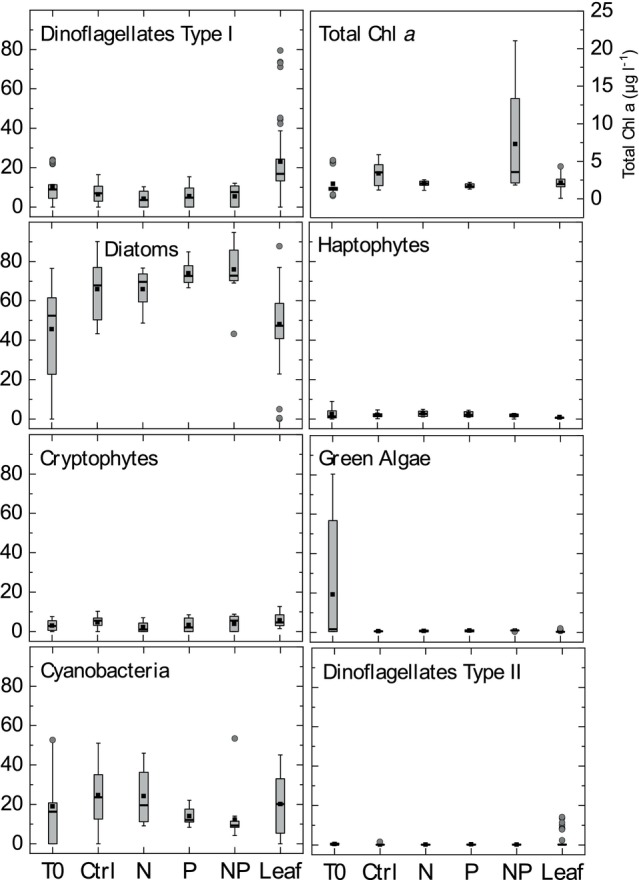
Boxplots showing treatment responses for algal groups. Means are indicated by small squares within the box, medians by a line within the box. The box indicates the first and third quartiles, and the bars show the 5th and 95th percentiles. Small circles represent outliers. *T*_0_ is the sample collected at the initiation of the experiment.

Algal groups showed diverse responses to the experimental manipulations in the bioassays (Figure 2). Diatoms and cyanobacteria were the largest contributors to the phytoplankton community. Percent abundances relative to the respective control varied widely, and within‐group variability was heterogeneous. Although some treatments showed relatively tight distributions, suggesting consistent responses, others had broader spreads, extreme values, and greater variability, suggesting that treatment effects were not uniform across all groups. However, dinoflagellate abundance increased notably in the leaf treatment. Total phytoplankton concentrations across all bioassays averaged 2.98 μg chlorophyll *a* · L^−1^ (*n* = 142, range = 0.09–21.06). The largest increases occurred in the N + P treatments, suggesting that N was the limiting factor for growth, with P as a minor co‐limiting factor.

**FIGURE 3:**
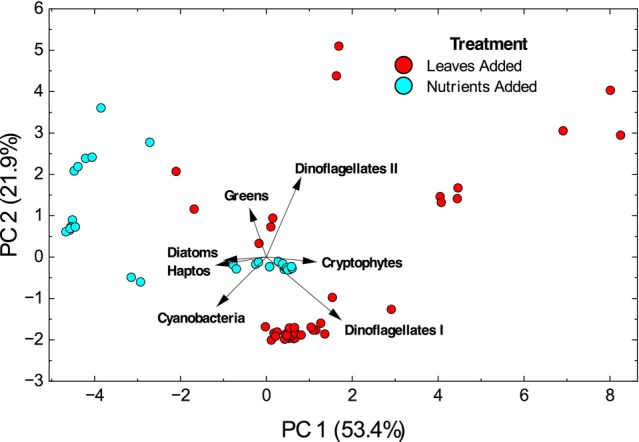
Principal component analysis (PCA) of phytoplankton community composition based on centered log‐ratio (CLR)‐transformed relative abundance data. The first two principal components explain 75.3% of the total compositional variance (PC1 = 53.4%, PC2 = 21.9%). Separation along PC1 reflects a gradient from non‐dinoflagellate‐dominated communities toward increased dinoflagellate dominance, with leaf additions shifting communities in this direction. The PCA highlights directional changes in relative dominance rather than discrete community clusters.

Analysis of phytoplankton community responses to the different treatments revealed significant effects (PERMANOVA, pseudo‐*F*_1,69_ = 15.137, *p* < 0.001), with treatment explaining 17.99% of the total compositional variance (*R* ^2^ = 0.1799, 95% bootstrap confidence interval = 0.139–0.253). Post hoc pairwise comparisons revealed significant differences for all treatment pairs, with the largest difference between leaf additions versus nutrient additions (pseudo‐*F*_1,69_ = 31.047, *R* ^2^ = 0.245, *p* = 0.0003), followed by control versus leaf additions (pseudo‐*F*_1,69_ = 12.811, *R* ^2^ = 0.122, *p* = 0.0003) and control versus nutrients (pseudo‐*F*_1,69_ = 6.066, *R* ^2^ = 0.065, *p* = 0.0018). Multivariate analysis using CLR‐PCA revealed changes in phytoplankton community composition across treatments (Figure 3). The first principal component (PC1) accounted for 53.4% of the total compositional variance, and the second principal component (PC2) accounted for 21.9%. Together, PC1 and PC2 explained 75.3% of the variance, showing that a low‐dimensional compositional gradient represents the main patterns in community composition. Principal component 1 exhibited a clear gradient in phytoplankton dominance. Negative scores along PC1 were associated with communities dominated by diatoms and cyanobacteria, whereas positive scores reflected increasing relative dominance of dinoflagellates, particularly type I dinoflagellates. Loadings indicated that diatoms and cyanobacteria contributed strongly (negative) to PC1, whereas type I dinoflagellates contributed positively. Type II dinoflagellates and cryptophytes contributed positively to a lesser extent. Principal component 2 captured secondary variation in community composition and explained an additional portion of the variance. The less dominant taxa (e.g., haptophytes, cryptophytes, and green algae) were less variable.

Treatment effects were most evident along PC1. Samples receiving leaf additions were consistently shifted toward higher PC1 scores relative to nutrient additions, indicating a pronounced shift toward dinoflagellate‐dominated community structure in samples receiving the senescent leaf treatments. Samples that received nutrient additions only (i.e., no leaves) occupied lower PC1 values, consistent with diatom‐dominated assemblages. The overall conclusion is that additions of senescent mangrove leaves shifted phytoplankton community composition toward increased dinoflagellate abundance, especially dinoflagellates type I.

**FIGURE 4:**
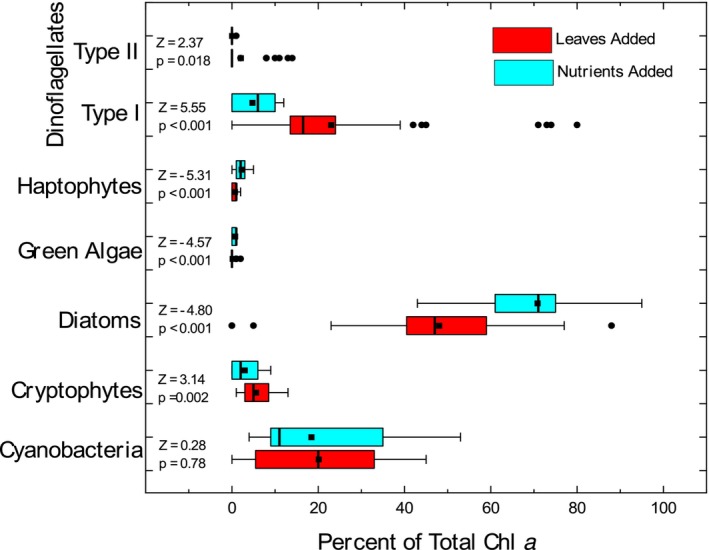
Boxplots showing algal group responses to the bioassay treatments. The respective results of statistical testing (Kruskal–Wallis ANOVA) for differences between treatments are indicated on the graph.

To further verify the PERMANOVA results and deliver a clear, easily understandable outcome., we evaluated the univariate responses for each algal group, expressed as the percentage of the control treatment for that group, using Kruskal‐Wallis ANOVAs (Figure 4). Consistent with the multivariate patterns, the univariate analyses showed a substantial, significant increase in the relative abundance of both dinoflagellate types (I and II) in leaf treatments compared with nutrient additions. In contrast, most other algal groups responded positively to N + P additions but negatively to leaf additions. No other major phytoplankton groups showed consistent treatment‐related changes, indicating that senescent mangrove leaf additions selectively promoted dinoflagellate dominance while seemingly inhibiting or minimally impacting the growth of other algal groups.

## DISCUSSION

Based on qualitative microscopy, Pinckney et al. (2018) reported that the most prevalent dinoflagellates in Mangrove Lagoon were species similar to *Alexandrium* and *Pyrodinium bahamense*. Other dinoflagellates present in smaller quantities included *Ceratium fusus*, *C. furca*, *C. lineatum*, *Prorocentrum emarginatum*, *Prorocentrum micans*, and *Protoperidinium quinquicorni*. Few diatoms were observed, mainly several *Skeletonema* species, alongside many benthic pennate diatoms, which exceeded 10^5^ cells · L^−1^. The most abundant diatom was *Pleurosigma* sp., dominating all other diatoms in the lagoon. Additionally, the heterotrophic dinoflagellate *Oxyrrhis marina* was detected in some samples.

The ambient DOC concentrations (prior to leaf addition) in the two bioassays differed markedly (79 vs. 212 μM C). Likewise, the C content of individual senescent mangrove leaves varied. We do not know the reason for the large difference, but we speculate it may be due to incubations in January versus May (winter vs. spring), when mangroves were actively growing. Thus, the DOC concentrations during the incubations were likely also different. We did not observe a fundamental difference in the overall phytoplankton responses between the two experiments, suggesting that DOC concentration was not a major factor. In addition, the responses we report were relative to the control, so all treatments within each bioassay had similar ambient DOC concentrations during incubation.

The PERMANOVA results revealed that nutrient and leaf additions significantly influenced phytoplankton community composition, with the treatment explaining about 18% of the multivariate variance. Bootstrap confidence intervals showed this effect was consistent and unlikely to be merely sampling noise. The most notable difference in community structure was between leaf and nutrient treatments, indicating that both factors can induce changes but elicit different responses. Therefore, the treatments represented moderate effect sizes typical in ecological studies.

Senescent mangrove leaf additions produced a consistent, directional shift in phytoplankton community structure toward increasing dinoflagellate relative abundance, suggesting that DOM inputs from mangrove leaf leachate, rather than inorganic nutrients (N and P) alone, are a primary driver of community reorganization and structure in Mangrove Lagoon. This response was selective rather than wholesale, with leaf additions enhancing dinoflagellate abundance without inducing broad changes in the relative abundances of other phytoplankton groups. In contrast, N and P nutrient additions (without leaves) primarily increased total biomass and maintained diatom‐dominated assemblages, consistent with N limitation but insufficient to change taxonomic dominance hierarchies. We interpret this pattern as evidence that senescent mangrove leaf leachate may enhance trophic pathways favorable to dinoflagellates, most likely by increasing the availability of DOC and/or dissolved organic N (DON; Moran et al., 1991; Muni‐Morgan et al., 2024; Zhang et al., 2025). An equally plausible explanation could be the selective inhibition of some algal groups by leaf leachate compounds such as proanthocyanidins, tannic acid, phenols, and humics (Alongi, 2014; Benner, Hatcher, et al., 1990; Benner, Weliky, et al., 1990; Maie et al., 2008; Zhang & Laanbroek, 2018; Zhou et al., 2023), which do not seem to inhibit dinoflagellates. In addition, bacterial decomposition of DOM may provide essential compounds such as vitamin B_12_ (cobalamin) required by dinoflagellates (Burkholder & Burkholder, 1958; Starr et al., 1957).

Type I dinoflagellates (those with the accessory pigment peridinin rather than fucoxanthin, which includes *Pyrodinium bahamense*) exhibited the strongest response to senescent leaf additions, suggesting functional specialization within the dinoflagellate community. Although pigment‐based analyses cannot resolve species‐level traits, the preferential response of this algal group implies a link between mangrove‐derived leachates and dinoflagellates adapted to complex or mixotrophic resource environments. Variability in how treatment responses occurred highlights the need to consider both the extent and the variability of changes within the community. Leaf addition treatments exhibited broader distributions, likely reflecting heterogeneity in leaf chemistry, microbial processing, and fine‐scale ecological interactions (Benner et al., 1986; Zhao et al., 2018). Such variability is ecologically meaningful and suggests that mangrove‐derived inputs elicit dynamic rather than uniform phytoplankton responses (Herrera‐Silveira & Ramírez‐Ramírez, 1996; Wan et al., 2018; Zhang & Laanbroek, 2018).

Compositional data analysis was critical for detecting these patterns (Egozcue et al., 2003). Centered log‐ratio principal component analyses revealed directional shifts in relative contribution along a continuous gradient rather than discrete community assemblages (Jolliffe, 2002). Ecologically, these findings have direct implications for the persistence of dinoflagellates and bioluminescent conditions in Mangrove Lagoon. Sustained mangrove leaf inputs and, by extension, the associated leachate may stabilize dinoflagellate abundances. Collectively, our results demonstrate that mangrove leaf‐derived OM, rather than inorganic nutrient enrichment, plays a central role in structuring phytoplankton communities in Mangrove Lagoon and potentially other biobays (Phlips et al., 2006; Pinckney et al., 2018; Seliger et al., 1971; Zimberlin, 2013). This study emphasizes the significance of OM quality and the use of compositional statistical analysis methods in detecting subtle but ecologically meaningful shifts in phytoplankton dominance.

Our results also suggest that leaf additions may alter phytoplankton community composition by selectively enhancing dinoflagellate relative abundances rather than by broadly restructuring the phytoplankton community. Although nutrient additions are commonly expected to stimulate phytoplankton growth (Reynolds, 2006), they did not produce comparable changes in relative community dominance, suggesting that N and P availability alone were insufficient to explain the increase in dinoflagellate relative abundances. The high variability in responses across algal groups underscores the difficulty of assessing the effects of the experimental treatments in the bioassays and highlights the importance of additional statistical approaches (i.e., CLR‐PCA) to detect shifts in community composition.

Our experiments were unable to determine the mechanism responsible for the enhanced dinoflagellate relative abundances in the senescent leaf treatments. Possible modes of action include selective inhibition of growth by leaf leachate in some algal groups, direct utilization of DOM by dinoflagellates, microbially mediated nutrient regeneration, allelopathic inhibition of competitors, shifts in grazing pressure, or micronutrient effects (e.g., iron, B_12_). More detailed experiments are needed to isolate these different potential effects. Although we did not demonstrate the exact mechanisms, our results indicate a direct effect (i.e., whether the mechanism is direct or indirect) of leaf additions on phytoplankton community composition.

We speculate that an increase in dinoflagellate relative abundances under leaf additions may reflect the effects of specific leachate compounds, enhanced availability of organic substrates, micronutrient conditions, or inhibition of other phytoplankton groups (Benner et al., 1986; Benner, Hatcher, et al., 1990; Benner, Weliky, et al., 1990; Herrera‐Silveira & Ramírez‐Ramírez, 1996; Zhao et al., 2018). Dinoflagellates are well known for their mixotrophic capabilities and competitive advantages under complex resource regimes, providing a plausible mechanism for the observed response (Mena et al., 2025; Mulholland et al., 2018; Zhang et al., 2025). Many peridinin‐containing dinoflagellates (type I) exhibit flexible nutritional strategies, ranging from strict autotrophy to facultative mixotrophy (Reynolds, 2006). Elevated concentrations of DOM (including amino acids, carbohydrates, and humics) derived from mangroves may increase the pool of bioavailable organic substrates in lagoon waters (Benner et al., 1986). Since dinoflagellates may either directly utilize OM or benefit from enhanced microbial regeneration and nutrient turnover using the added OM (Dagenais‐Bellefeuille & Morse, 2013; Yang et al., 2024), the extent to which dinoflagellates use mangrove leachate as a DOM nutritional source affects both trophic processes and biogeochemical cycling in these lagoons.

Our bioassays represented short‐term (48 h) responses to nutrient and leaf additions and therefore showed community responses to a “pulsed” event. Our results did not allow us to evaluate the impacts of long‐term exposures. Nonetheless, the pulsed nutrient addition bioassays indicate that the initial responses of the total phytoplankton community (chlorophyll *a*) and the chlorophytes, cryptophytes, cyanobacteria, diatoms, and haptophytes were co‐limited by N and P. Dinoflagellates, however, did not respond to the nutrient additions, suggesting that N and P were not a major determinant of relative abundances. A comparison of initial and final dinoflagellate concentrations in the bioassays indicates that the incubation period was sufficient to detect significant growth responses. In contrast to other studies (Badylak et al., 2007; Fan et al., 2003; Phlips et al., 2006; Sellner et al., 2003), nutrient enrichment did not stimulate dinoflagellate growth in our study. Our results, however, are consistent with the recent suggestion by Usup et al. (2012) that some tropical dinoflagellates are not competitive for nutrients and will not become dominant under high N and P conditions.

Dissolved organic matter is an important nutritional source for dinoflagellates, and mangrove leaves leach relatively large amounts of labile DOM, along with sugars, proteins, polyphenols, and inorganic nutrients, into the surrounding waters (Benner et al., 1986; Benner, Hatcher, et al., 1990; Benner, Weliky, et al., 1990; Maie et al., 2008; Zhang & Laanbroek, 2018). Although DOM may provide nutrition for dinoflagellates, mangrove DOM may also inhibit the growth of other phytoplankton (Doblin et al., 1999; Stolte et al., 1994). In our study, all phytoplankton groups except dinoflagellates were negatively affected by the addition of mangrove leaves. This response supports recent work by Muni‐Morgan et al. (2024), who showed that bioassay treatments amended with DOM‐rich stormwater runoff supported *Pyrodinium bahamense* growth, particularly through drawdown of reduced N‐forms (dissolved organic N and ammonium). Thus, DOM inputs via senescent mangrove leaves appear to inhibit or minimally impact all phytoplankton groups except dinoflagellates. Collectively, these results indicate that phytoplankton community composition and abundances may be controlled by a balance between low‐nutrient concentrations and mangrove OM input in this lagoon. Under ambient conditions of low‐nutrient availability and high DOM concentrations, dinoflagellates may have a slight competitive advantage over other phytoplankton.

One caveat of our conclusions is that the bioassays simulated acute inputs of relatively large amounts of senescent leaf material, and therefore, the community responded to a “pulsed” event rather than chronic, long‐term exposures. Thus, the leaf‐derived inputs can rapidly shift the competitive balance in the phytoplankton community, but the effects of long‐term stabilization of community structure remain to be determined. Since the incubation period (48 h) is short compared to the growth rates of many dinoflagellates, such as *Pyrodinium bahamense*, the observed increases in their relative abundance probably indicate quick changes in community composition rather than complete population doubling. These shifts could result from varied responses among different taxa, such as faster growth among some dinoflagellates, mixotrophic activity, or the suppression of competing phytoplankton groups in conditions influenced by leaf‐derived organic matter.

Other studies suggested that slow growth rates and high nutrient loading may preclude dinoflagellate growth (Phlips et al., 2006; Usup et al., 2012). Conversely, a restricted or episodic supply of new inorganic nutrients, combined with large size and motility, may enable dinoflagellates to search for and store nutrients in more stable water columns (Phlips et al., 2006; Reynolds, 2006; Usup et al., 2012). As in similar systems, we suspect that long water residence time and calm conditions in Mangrove Lagoon further contribute to a favorable environment for dinoflagellate growth, including that of *Pyrodinium bahamense* (Margalef, 1978; Seliger et al., 1970, 1971; Sullivan & Swift, 2003).

## CONCLUSIONS

Using compositional data analysis and CLR‐PCA, we showed that additions of senescent mangrove leaves consistently altered phytoplankton community structure by shifting the relative contribution of algal groups toward dinoflagellates, particularly peridinin‐containing dinoflagellates. In contrast, inorganic nutrient additions (N and P) did not produce similar effects. These results underscore the importance of organic matter inputs (particularly mangrove leaf leachate) over inorganic nutrients alone in shaping phytoplankton community composition. More broadly, this analysis highlights the value of compositional approaches (CLR‐PCA) for detecting subtle yet robust ecological responses that traditional analyses of relative abundance data may obscure. Future experiments that chemically partition the filtered leachate from senescent leaves would allow mechanistic resolution of direct versus microbially mediated pathways and identify specific compound effects on dinoflagellate abundance regulation.

## AUTHOR CONTRIBUTIONS

**James L. Pinckney:** Conceptualization (lead); data curation (supporting); formal analysis (lead); funding acquisition (lead); investigation (equal); methodology (equal); project administration (lead); resources (equal); supervision (lead); validation (lead); visualization (lead); writing – original draft (lead); writing – review and editing (lead). **Michelle Zimberlin:** Formal analysis (equal); investigation (equal); writing – review and editing (supporting). **Dianne I. Greenfield:** Investigation (equal); writing – review and editing (supporting).
